# Femoral Arterial Perfusion Guided by Intraoperative Transesophageal Echocardiography for Mesenteric Malperfusion in Acute Type A Aortic Dissection

**DOI:** 10.3400/avd.oa.26-00006

**Published:** 2026-04-11

**Authors:** Chihaya Ito, Go Kuwahara, Hiromitsu Teratani, Yuichi Morita, Yuta Sukehiro, Masato Furui, Hideichi Wada

**Affiliations:** Department of Cardiovascular Surgery, Fukuoka University, Fukuoka, Fukuoka, Japan

**Keywords:** aortic dissection, malperfusion, visceral ischemia

## Abstract

**Objectives:**

This study evaluated the outcomes of acute type A aortic dissection (ATAAD) complicated by mesenteric malperfusion managed with femoral arterial perfusion and intraoperative transesophageal echocardiography (TEE).

**Methods:**

We retrospectively reviewed 246 patients who underwent surgery for ATAAD between April 2011 and May 2022. Mesenteric malperfusion was identified in 8 of 49 patients (20%) with malperfusion syndrome. Femoral arterial perfusion was initiated to restore true lumen flow, and intraoperative TEE was used to assess abdominal aortic true lumen expansion and superior mesenteric artery (SMA) perfusion. Central aortic repair was performed after confirmation of mesenteric reperfusion.

**Results:**

Dynamic and static mesenteric obstructions were present in 5 and 3 patients, respectively. Femoral perfusion improved SMA flow in all dynamic cases and in 2 static cases. Seven patients (87.5%) survived to discharge; 1 patient with preoperative coma, shock, and static SMA obstruction died. In 1 patient with combined SMA and celiac artery obstruction, residual celiac malperfusion was not detected intraoperatively.

**Conclusions:**

Femoral arterial perfusion guided by intraoperative TEE is a feasible and effective strategy for mesenteric malperfusion in ATAAD. However, static branch obstruction and limitations of TEE warrant consideration of hybrid approaches.

## Introduction

Acute type A aortic dissection (ATAAD) remains one of the most fatal cardiovascular emergencies,^1)^ and the presence of malperfusion syndrome further complicates its management and significantly worsens outcomes.^2–5)^ Among its various manifestations, mesenteric malperfusion is one of the most devastating. According to the International Registry of Acute Aortic Dissection (IRAD), mesenteric malperfusion—occurring in approximately 3.7% of ATAAD cases—is often clinically subtle and difficult to detect. Furthermore, patients with mesenteric involvement demonstrate a markedly higher in-hospital mortality rate (63.2%) compared with those without mesenteric ischemia (23.8%; p <0.001).^6)^

Despite the increasing recognition of its prognostic significance, an optimal therapeutic strategy for mesenteric malperfusion in ATAAD is yet to be established. Medical management or isolated endovascular interventions have been associated with poor outcomes, whereas several studies have reported improved survival with surgical or hybrid approaches.^6,7)^ However, owing to diagnostic challenges, heterogeneous presentations, and variations in institutional expertise, treatment strategies vary widely among centers.

At our institution, perfusion strategies are tailored to the mechanisms underlying malperfusion. Although ascending aortic cannulation is routinely used for ATAAD repair, in patients presenting with malperfusion syndrome, distal perfusion is preferentially employed to enhance true lumen flow. Adjunctive or exclusive femoral arterial perfusion is the standard approach in cases of mesenteric malperfusion. Intraoperatively, transesophageal echocardiography (TEE) is used to evaluate abdominal aortic true lumen expansion and improve superior mesenteric artery (SMA) flow, whenever feasible. Moreover, because a significant proportion of ATAAD surgeries at our institution are performed during nighttime or emergent conditions, revascularization- or laparotomy-first strategies are rarely pursued because of constraints regarding logistics and personnel.

The absence of consensus regarding optimal management and the limited availability of data describing real-world institutional strategies warrant further investigation. Therefore, this study aimed to retrospectively assess the outcomes of patients with ATAAD complicated by mesenteric malperfusion at our institution, examine the validity of our management strategy, and identify areas for potential improvement.

## Materials and Methods

### Study design and patient population

**Fig. 1:**
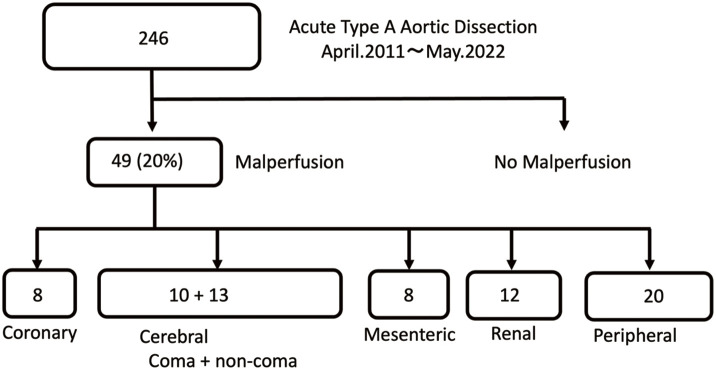
Patient flow diagram.

This retrospective cohort study included 246 consecutive patients who underwent surgical repair for ATAAD at our institution between April 2011 and May 2022. Patients were stratified according to the presence or absence of malperfusion syndrome. The study design and patient selection are shown in **Fig. 1** as a flowchart.

The institutional review board of Fukuoka University Hospital (U22-11-012; 11/17/2022) approved the study protocol and waived the requirement for informed consent owing to the retrospective nature of the study.

### Preoperative assessments

All patients underwent comprehensive preoperative evaluations. Contrast-enhanced computed tomography (CT) scans of the chest, abdomen, and pelvis were performed to delineate the extent of the dissection and identify organ ischemia. Aortic valve morphology and ventricular function were assessed using transthoracic echocardiography and TEE, respectively. Laboratory tests included the complete blood count, serum biochemistry, coagulation parameters, and arterial blood gas analysis. Neurological consultations were conducted for patients with suspected cerebral ischemia.

### Definitions and classifications

All patients diagnosed with ATAAD were included in the initial cohort. ATAAD was defined as a dissection involving the ascending aorta, regardless of the location of the primary entry tear, as per the Stanford classification.

Malperfusion syndrome was defined as the presence of clinical, biochemical, or imaging evidence of end-organ ischemia secondary to aortic dissection. Mesenteric malperfusion was diagnosed based on a combination of clinical findings (e.g., abdominal pain), biochemical abnormalities (elevated serum lactate), and imaging evidence of compromised SMA flow or severe true lumen compression on CT. In patients with abdominal symptoms and elevated lactate levels but without definitive SMA occlusion on CT, intraoperative TEE assessment was used to determine functional flow limitation.

**Fig. 2:**
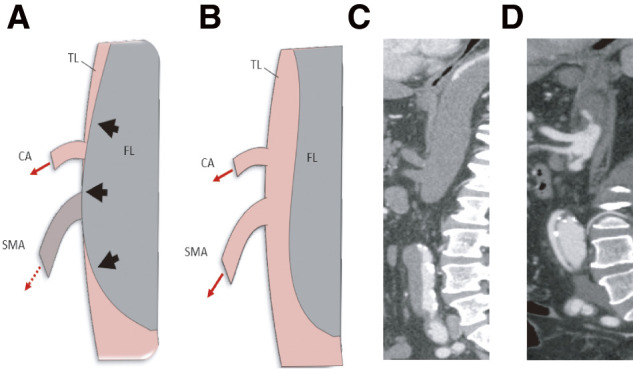
(**A**, **B**) Schematic illustration of dynamic obstruction in mesenteric malperfusion. (**C**, **D**) Representative CT images before and after surgery, respectively. TL: true lumen; FL: false lumen; CA: celiac artery; SMA: superior mesenteric artery

Dynamic obstruction was defined as compression of the SMA true lumen by the intimal flap without fixed branch vessel occlusion on contrast-enhanced CT, in which flow limitation was presumed to be caused by pressure imbalance between the true and false lumens. A schematic diagram and representative CT images of SMA dynamic obstruction are shown in **Fig. 2**.

Static obstruction was defined as fixed narrowing or occlusion of the SMA due to extension of the dissection flap into the branch vessel, thrombosis, or radiographically apparent branch vessel compromise that was unlikely to resolve solely with restoration of true lumen pressure.

### Surgical procedures

All surgeries were performed via median sternotomy under general anesthesia and cardiopulmonary bypass. The surgical strategy was individualized according to the extent of dissection and patient-specific anatomy.

Arterial cannulation sites included the axillary artery (n = 14), ascending aorta using the Seldinger technique (n = 27), left ventricular apex (n = 3), and femoral artery (n = 22). Dual-site arterial cannulation was performed in 17 patients to optimize systemic perfusion.

Hemiarch replacement (n = 38, 78%) was performed when the primary entry tear was confined to the ascending aorta and proximal arch. Conversely, total arch replacement (n = 11, 22%) was performed when the primary entry was located distal to the arch or in low-risk younger patients, including those with Marfan syndrome.

### Management strategy for mesenteric malperfusion

Our institutional approach to mesenteric malperfusion followed a stepwise protocol.

1. Initiation of femoral arterial perfusion to increase true lumen pressure.
2. Intraoperative evaluation of abdominal aortic true lumen expansion and mesenteric perfusion using TEE.
3. Central aortic repair to restore antegrade true lumen perfusion.
4. Post-repair reassessment of aortic and mesenteric perfusion using TEE before chest closure.

Regardless of whether intestinal ischemia resulted from dynamic or static obstruction, enlargement of the true lumen of the abdominal aorta and improvement in SMA perfusion on TEE prompted central aortic repair. Using TEE, visualization was generally limited to the abdominal aortic true lumen at the level of the SMA origin and the proximal 1–2 cm of the SMA. Distal SMA branches could not be reliably evaluated. True lumen expansion was defined as an increase in true lumen cross-sectional area at the level of the SMA origin, occupying more than 50% of the total aortic lumen compared with the pre-perfusion status. Improvement in SMA flow was defined as the appearance or augmentation of antegrade Doppler flow signals compared with baseline findings before femoral arterial perfusion. Intraoperative TEE assessment of SMA perfusion was not feasible in all patients. In particular, visualization may be limited by anatomical factors such as insufficient gastric distension, overlying bowel gas, or unfavorable probe positioning. In addition, operator experience plays an important role in obtaining adequate imaging. Depending on the anesthesiologist’s level of expertise, we also performed evaluations using pulsed-wave Doppler; however, this was not possible in all cases, and we were unable to set a consistent blood flow velocity. Gain settings were adjusted to optimize signal detection. No complications related to TEE probe manipulation, including esophageal or gastric injury, were observed in this series.

### Data collection and statistical analysis

Clinical data, operative variables, and postoperative outcomes were extracted from electronic medical records. Continuous variables are expressed as mean ± standard deviation, whereas categorical variables are expressed as frequencies and percentages. Mortality was defined as in-hospital death or death within 30 days of surgery. Statistical analyses were performed using JMP version 14.0 (SAS Institute, Cary, NC, USA). Statistical significance was set at p <0.05.

## Results

### Patient demographics and surgical factors

Of the 246 patients who underwent ATAAD repair, 49 (20%) presented with malperfusion syndrome. The mean age was 65.8 ± 12.6 years, and 63% were male. The majority had DeBakey type I dissection (94%), with the primary entry located in the ascending aorta in most cases. Hemiarch replacement cases accounted for 78% of the procedures, a higher proportion than total arch replacement. As for concomitant procedures, there were 2 cases (4%) of aortic root replacement, 4 cases (8%) of aortic valve replacement, 8 cases (16%) of coronary artery bypass grafting, and 2 cases (4%) that required femoral–femoral artery bypass. The mean operation time was 401 mins (range: 336–520), the mean circulatory arrest time was 52 mins (range: 44–65), and the mean blood loss was 624 ml (range: 351–1098).

### Distribution of malperfusion sites

In 49 patients, malperfusion syndrome was classified as shown in **Table 1**. Of the 23 patients with cerebral malperfusion, 10 (4.1%) were comatose. Multi-organ malperfusion occurred frequently; only 17 patients had involvement of a single site, 22 had 2 affected sites, and 10 presented with malperfusion in 3 or more sites.

**Table 1:** Characteristics of malperfusion cases presenting at our hospital

Malperfusion	Cases (n = 49)	Death (n = 13, %)
Coronary artery	8	5 (63%)
Cerebral vessels (coma)	10	7 (70%)
Cerebral vessels (non-coma)	13	2 (15%)
Mesenteric	8	1 (13%)
Renal	12	3 (25%)
Peripheral	20	1 (5%)

### Mortality according to the site of malperfusion

The overall mortality among patients with malperfusion was 27%, with substantial variation depending on the affected organ system. Mesenteric malperfusion was associated with a mortality rate of 13%, which was lower than previously reported rates in the literature.^6)^

### Clinical features and outcomes of mesenteric malperfusion

Of the 246 patients, 20 patients (8%) had retrograde type A dissection originating from a distal entry tear. However, none of the 8 patients with mesenteric malperfusion had retrograde type A dissection due to a primary type III lesion. Furthermore, in all 8 mesenteric cases, intraoperative TEE confirmed preferential true lumen perfusion after femoral arterial perfusion, and no evidence of predominant false lumen perfusion was observed. Among the 8 patients with mesenteric malperfusion, only 2 presented without concomitant malperfusion in other organ systems. Renal malperfusion was observed in 4 patients, lower-limb malperfusion in 3, and cerebral malperfusion in 3, 1 of whom presented in a comatose state. The mechanisms of mesenteric malperfusion included dynamic obstruction in 5 patients and static obstruction in 3. Femoral arterial perfusion was used in all cases, with adjunctive cannulation of the ascending aorta in 5 patients and that of the axillary artery in 2. Surgical procedures included total arch replacement in 3 patients and hemiarch replacement in 2. The median interval from symptom onset to surgery was 7.0 h (5.6–9.8). Details of all mesenteric malperfusion cases are shown in **Table 2**.

**Table 2:** Characteristics of mesenteric malperfusion cases

Cases	Age	Sex	Other malperfusion	Mechanism	Perfusion	TEE finding SMA flow (±)			Procedure
						Initial	After FA perfusion	End of surgery	
1	74	F	Right kidney	Dynamic	FA+Ascd	Flow(−)	Flow(+)	Flow(+)	HAR
2	62	F	Left kidney, right lower limb	Dynamic	FA+Ascd	Flow(−)	Flow(+)	Flow(+)	TAR+ET
3	79	M	Left kidney, left lower limb, brain	Static	FA+Ascd	No detect	No detect	No detect	HAR
4	64	M		Dynamic	FA+Ascd	Flow(−)	Flow(+)	Flow(+)	TAR+FET
5	68	M	Left kidney, brain	Static	FA+Ax	Flow(−)	Flow(+)	Flow(+)	HAR+Bentall
6	52	M	Brain	Dynamic	FA+Ax	Flow(−)	Flow(+)	Flow(+)	HAR
7	44	M		Dynamic	FA	Flow(−)	Flow(+)	Flow(+)	TAR
8	80	M	Left kidney, brain (coma), lower limb	Static	FA+Ascd	No detect	No detect	No detect	HAR

Ascd: ascending aorta; Ax: axillary artery; FA: femoral artery; FET: frozen elephant trunk; HAR: hemiarch replacement; TAR: total arch replacement; TEE: transesophageal echocardiography; SMA: superior mesenteric artery

CT demonstrated concomitant celiac artery (CA) malperfusion in 3 patients. However, intraoperative assessment of CA flow using TEE was not consistently feasible due to technical limitations. In patient 3, who had a static obstruction, enlargement of the true lumen in the abdominal aorta was confirmed by TEE after establishing cardiopulmonary bypass; however, SMA flow could not be adequately evaluated, prompting laparotomy to directly confirm the restoration of mesenteric perfusion. Seven patients (87.5%) remained alive at hospital discharge. One patient (12.5%) died of disseminated intravascular coagulation and multi-organ failure. The fatal case (patient 8) involved static SMA obstruction with preoperative coma and shock, representing an extreme salvage-type presentation, and a detailed TEE evaluation of the SMA was not performed.

**Fig. 3:**
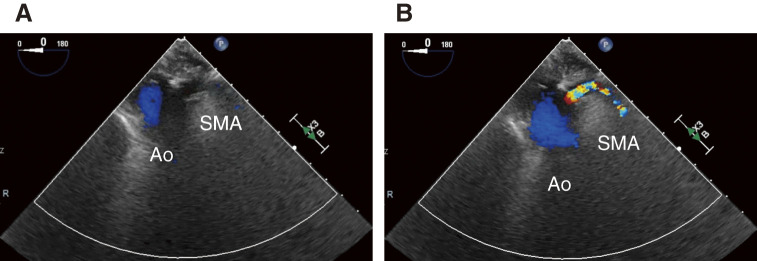
(**A**, **B**) Representative TEE images before and after FA perfusion, respectively. Ao: aorta; SMA: superior mesenteric artery

In Patient 5, who had concomitant celiac and SMA malperfusion, TEE confirmed true lumen expansion and improved SMA flow after femoral arterial perfusion. Intraoperative TEE images before and after FA perfusion in this case are shown in **Fig. 3**. This finding enabled the surgical team to proceed with the Bentall procedure and hemiarch replacement. Although the postoperative course was uneventful, contrast-enhanced CT subsequently demonstrated persistent CA occlusion, suggesting that femoral arterial perfusion alone may be insufficient to restore blood flow in cases of static visceral artery obstruction.

## Discussion

Mesenteric malperfusion represents an exceptionally severe condition, with a prognostic impact comparable with that of coronary malperfusion or profound cerebral malperfusion.^6–8)^ In recent years, several management strategies have emerged as viable alternatives to the traditional central repair–first approach,^9)^ including revascularization-first strategies aimed at restoring mesenteric perfusion before aortic repair,^10,11)^ and laparotomy-first approaches in patients with suspected bowel necrosis.^12)^ In this study, we demonstrated that femoral arterial perfusion combined with intraoperative TEE assessment is a practical and effective strategy for managing mesenteric malperfusion in ATAAD. This approach enabled real-time evaluation of true lumen expansion and SMA flow, facilitating appropriate decision-making regarding the timing of central repair.

TEE is a useful modality for assessing mesenteric malperfusion.^13,14)^ At our institution, intraoperative evaluation of abdominal aortic true lumen expansion and SMA flow using TEE is routinely performed in the management of patients with malperfusion syndrome. For patients presenting with mesenteric malperfusion, the fundamental approach is to initiate arterial perfusion distal to the lesion, with adjunctive or exclusive femoral arterial perfusion being the standard strategy. Following the establishment of cardiopulmonary bypass, TEE was performed to evaluate the degree of true lumen expansion and improvement in SMA flow. The CA may play an important role in maintaining mesenteric perfusion through collateral pathways with the SMA. Therefore, even in cases where direct visualization of the SMA is limited, assessment of CA flow and overall true lumen expansion may provide indirect evidence of improved mesenteric circulation. Furthermore, distal SMA occlusion cannot be reliably detected using TEE alone. Adjunctive modalities, such as surface ultrasonography to evaluate bowel peristalsis or the presence of ascites, may provide additional information regarding intestinal viability, particularly in cases where distal ischemia is suspected.

If reperfusion was inadequate, the strategy was escalated to laparotomy or endovascular intervention. Before chest closure, TEE was performed again to confirm resolution of mesenteric malperfusion.

In cases of dynamic SMA obstruction, the restoration of true lumen flow through FA perfusion is theoretically expected to improve mesenteric perfusion. Corroborating this notion, all 5 patients with dynamic obstruction in our series showed improved SMA flow after femoral arterial perfusion, which enabled definitive central repair and yielded favorable clinical results. **Figure 2** shows the preoperative and postoperative schematic diagrams and representative CT images for a case of dynamic obstruction.

Interestingly, 2 of 3 patients with static obstruction demonstrated improved SMA flow after femoral arterial perfusion. Although static obstruction is traditionally considered less responsive to perfusion strategies, several mechanisms may explain this observation. Increased true lumen pressure may reduce superimposed dynamic compression, restore partial luminal patency in cases of incomplete thrombosis, or alter branch vessel geometry by decreasing false lumen pressure. Nevertheless, femoral arterial perfusion alone cannot be considered a definitive solution for static malperfusion. In 1 of these patients, TEE could not definitively confirm SMA reperfusion, and laparotomy was performed to directly evaluate the mesenteric flow using Doppler assessment. The only mortality occurred in a patient presenting in an extremely critical state with mesenteric ischemia complicated by coma and preoperative shock. In this salvage-type situation, a rapid lifesaving intervention took precedence over detailed evaluation of SMA perfusion; accordingly, central repair was performed as the initial step.

Our institutional strategy of initiating femoral perfusion followed by TEE-based evaluation of visceral perfusion has several advantages. It does not require specialized equipment and can be implemented using a standard setup for ATAAD surgery. Moreover, it imposes minimal additional burden during nighttime or emergency procedures, making it practical for institutions without ready access to hybrid operating rooms or endovascular specialists. This approach may also help avoid unnecessary endovascular interventions or exploratory laparotomies in select patients.

However, femoral arterial perfusion alone cannot reliably restore visceral perfusion in all cases, and TEE-based assessments may be insufficient in certain situations. Herein, in 1 patient with concomitant SMA and CA occlusion, the SMA flow improved with femoral arterial perfusion; however, postoperative CT revealed persistent CA occlusion that was not detected intraoperatively. This case illustrates that femoral arterial perfusion alone may be inadequate for resolving static visceral branch obstruction. Although the postoperative course was uneventful, a similar undetected obstruction involving the SMA may have been fatal.

Hybrid operating rooms offer direct angiographic assessment of visceral branch perfusion, including SMA flow, after cardiopulmonary bypass initiation. This is the most reliable method of evaluating mesenteric reperfusion. Furthermore, hybrid facilities enable a seamless transition to endovascular intervention when femoral arterial perfusion alone fails to restore adequate flow. The benefits of performing ATAAD in a hybrid operating room have been reported previously.^10,15,16)^

In the present study, our strategy of combining femoral arterial perfusion and TEE-based assessments appeared to be a reasonable and acceptable approach for managing mesenteric malperfusion. However, mesenteric ischemia is a complex condition influenced not only by the underlying mechanism (dynamic vs. static) but also by factors such as symptom duration and the overall hemodynamic status.^17,18)^ Therefore, FA perfusion alone cannot be expected to resolve all cases of mesenteric malperfusion. Accordingly, the optimal management of mesenteric ischemia requires a comprehensive assessment of these factors, with individualized therapeutic strategies tailored to the specific pathophysiology and clinical condition of each patient.

### Limitations

This study has several limitations. First, this was a single-center retrospective analysis with a relatively small sample size, particularly for patients with mesenteric malperfusion, which limited the generalizability of the findings. Second, the assessment of mesenteric reperfusion relied primarily on intraoperative TEE, which may not reliably detect persistent CA or SMA obstruction, especially in cases of static malperfusion. Furthermore, it depends on the anesthesiologist's expertise in TEE. Third, because endovascular- or laparotomy-first strategies are rarely performed at our institution for logistical reasons, a direct comparison with alternative management strategies was not possible. Finally, unmeasured clinical variables, including the variations in the hemodynamic status, may have influenced the outcomes.

## Conclusion

Our institutional strategy of establishing femoral arterial perfusion and assessing mesenteric reperfusion using intraoperative TEE may offer a practical and effective approach for managing mesenteric malperfusion in ATAAD, achieving better outcomes than those previously reported. However, our findings suggest that femoral arterial perfusion alone cannot fully resolve all patterns of visceral ischemia, particularly those involving static obstruction. Individualized treatment and the incorporation of more precise intraoperative imaging, such as hybrid operating room angiography, may further improve outcomes in this high-risk population.
